# Treg Heterogeneity, Function, and Homeostasis

**DOI:** 10.3389/fimmu.2019.03100

**Published:** 2020-01-14

**Authors:** Daniil Shevyrev, Valeriy Tereshchenko

**Affiliations:** Research Institute for Fundamental and Clinical Immunology (RIFCI), Novosibirsk, Russia

**Keywords:** T-regulatory cell, single-cell analysis (SCA), TCR—T cell receptor, antigen-specific activation, immune equilibrium, TCR repertoire

## Abstract

T-regulatory cells (Tregs) represent a unique subpopulation of helper T-cells by maintaining immune equilibrium using various mechanisms. The role of T-cell receptors (TCR) in providing homeostasis and activation of conventional T-cells is well-known; however, for Tregs, this area is understudied. In the last two decades, evidence has accumulated to confirm the importance of the TCR in Treg homeostasis and antigen-specific immune response regulation. In this review, we describe the current view of Treg subset heterogeneity, homeostasis and function in the context of TCR involvement. Recent studies of the TCR repertoire of Tregs, combined with single-cell gene expression analysis, revealed the importance of TCR specificity in shaping Treg phenotype diversity, their functions and homeostatic maintenance in various tissues. We propose that Tregs, like conventional T-helper cells, act to a great extent in an antigen-specific manner, which is provided by a specific distribution of Tregs in niches.

## Introduction

As adaptive immunity evolved, there emerged a specialized immune response regulation system. Cartilaginous fish that appeared over 450 million years ago developed a thymus as well as the orthologous genes of key cytokines and transcription factors of the main lymphocyte subpopulations (1); however, whether this class of organisms had a fully developed adaptive immunity and a specific regulation system is debatable. It took some 36 million more years for full-fledged adaptive immunity to emerge, when bony fish appeared (2, 3). A number of aromorphoses, including the secondary immune response, gave bony fish a significant competitive advantage and directed the further evolution of this group of organisms. Despite different subpopulations of effector cells being able to suppress each other (4–7), there emerged a special subpopulation of T cells capable of specific immune response regulation. As such, these lymphocytes were named T regulatory cells, or Tregs. Phylogenesis identifies these lymphocytes in bony fish first, making them the most ancient organisms proven to have basic populations of effector T cells (3). Note that the ontogenetic Treg development in these organisms is linked to the thymus. Further Treg evolution was seemingly associated with the emergence of placental mammals that were the first to feature peripheral induction of Tregs (pTregs) from the effector precursors; cells carrying αβ-TCR and the CD4 co-receptor are hereinafter referred to as Tconv. It is assumed that such pTregs were not only able to control placental immunity but also gained greater involvement in regulating the adaptive immune response along with thymic Tregs (tTregs) (8).

Treg research is of interest when it involves the pathogenesis and therapy of various autoimmune diseases, cancers, and allergies, as well as transplantation studies and tissue engineering. The cells have extremely broad functionality; they ensure tolerance to autoantigens (9) and to the antigens of commensal microflora (10), they limit excess immune response, induce tolerance to food antigens (11), regulate the fetoplacental immunity (12), and play a role in the homeostasis and regeneration of various tissues (13–16). Treg research began more than 40 years ago. As early as the 1970s, Gershon and Kondo experimented on mice with removed thymi and were the first to suggest the existence of suppressor T lymphocytes (17). In the years that followed, great efforts were made to identify and study this population of T cells. Thus, eliminating some subpopulations of T lymphocytes in mice triggered an autoimmune syndrome that affected various organs and tissues, which indirectly proved the existence of a specific T cell population that provides peripheral autotolerance (18). However, studies into suppressor immunity were impeded by a lack of phenotypic Treg markers, the diversity of suppressor mechanisms, and the difficulty of obtaining antigen-specific Treg clones for more subtle cellular and molecular analysis. It was only in the 1990s that the prerequisites for further Treg studies were fulfilled, as there appeared transgenic animals and monoclonal antibodies, and scientists identified the primary suppressive cytokines (IL-10 and TGF-β) as well as their producers (19–21). Nevertheless, defining the Treg phenotype remained an extremely important problem until the early 2000s, i.e., until researchers found that IL-2 and its CD25 receptor were critical to developing and maintaining a Treg pool (22, 23). At the same time, scientists discovered the main transcription factor of these cells, FoxP3, which, as discovered later, enabled Tregs to function and was associated with high CD25 expression (24–26). Thus, the Treg phenotype was defined as CD3^+^CD4^+^CD25^hi^FoxP3^+^; however, that FoxP3 requires intracellular staining and that CD25 is also a marker of activated Tconv limited the applicability of this phenotype to cell research. In the following years, researchers found a negative correlation of IL-7 receptor (CD127) expression and FoxP3 expression, characterizing Tregs as low CD127 expression cells (27). However, even this factor is not universal because its expression from Tconv may be reduced under certain circumstances, e.g., when affected by IL-7 or other common γ-chain cytokines (28). However, CD3^+^CD4^+^CD25^hi^CD127^lo^ is currently the most common phenotype, especially when the Treg population is isolated by means of FACS or immunomagnetic separation with further evaluation of FoxP3 expression being possible. Further Treg research identified additional phenotypic markers, mainly related to Treg functions.

## Treg Heterogeneity

As noted earlier, Tregs may develop ontogenetically in the thymus (tTregs), as well as and peripherally (pTregs) from effector cells. tTregs express FoxP3 constitutively and have a T cell receptor (TCR) of relatively high autoaffinity. These cells are predominant in the bloodstream and in the lymph nodes; they are mainly involved in providing tolerance to autoantigens (9). Peripherally, CD4^+^-effector cells affected by IL-2 and TGF-β may under certain conditions begin to express FoxP3, thus becoming functional equivalents of tTregs (29). Such pTregs are most common in the peripheral barrier tissue and are mainly involved in preventing local inflammation in the presence of exogenous antigens. It is well-known that Tregs cells and naive CD4^+^ Tconv cells have non-overlapping TCR repertoires, a small percentage of equal affinity TCRs are found in both CD4^+^ and Treg cell populations (30). So therefore, the TCR repertories of tTregs cells and pTregs cells have been shown toare be distinct: in the tTreg cell TCR repertoire is biased toward self-recognition, and TCRs expressed in pTregs cells can recognize foreign antigens with high affinity (31). This is has been well-confirmed by analysis of amino acid CDR3 TCR repertoire overlaps, which revealed separate clusterings of Tconv cells and Tregs cells (32). Furthermore, CDR3s containing strongly interacting amino acids are more prominent in the Treg cell TCR pool compared with Tconv cells (33). That is consistent with previously obtained data about the higher TCR affinity of Tregs for self-peptide–MHC complexes? Thus, the TCR specificity spectra of tTregs and pTregs barely overlap. This may be due to the requirement in additional Tregs with specificity to antigens, which are not presented in the thymus by dint of AIRE or Fezf2, such as innocuous environmental antigens. These differences in the tTreg TCR pool from that of Tconv cells is determined during thymic selection based on the strength of the TCR signal (34) and a high TCR Treg affinity to self-antigens, which may enable Treg precursors to compete more efficiently for the limited niche of thymic antigen-presenting cells (33, 35).

It is worth noting that human tTreg- and pTreg-specific markers have not been discovered thus far. The high expression of Helios and Neuropilin-1 in mice suggests a thymic origin (36–38). What distinguishes tTregs and pTregs is the stability of FoxP3 expression in different settings. It has been found that FoxP3 expression by pTregs is transient in nature; in the case of inflammation, pTregs can differentiate into exFoxP3 effector cells that have the phenotype of Th-17 lymphocytes (RORyt^+^) (39), which are pathogenic for autoimmune disease-affected patients (40, 41). Normally, this transition is observed, for instance, in the intestines, which contain FoxP3^+^RORyt^+^ Treg lymphocytes associated with mucosa-associated lymphoid tissue functions (42). The stability of FoxP3 expression greatly depends on the methylation of CpG islets in the locus of the second intron enhancer in the FoxP3 gene, which is also referred to as the conservative non-coding sequence 2 (CNS2) or Major TSDR (Treg-specific demethylated region). CNS2 demethylation stabilizes FoxP3 expression and is characteristic of tTregs (43–45). Attempts are under way to artificially stabilize FoxP3 expression in *in vitro* induced Tregs for further clinical application (46).

**Figure 1:**
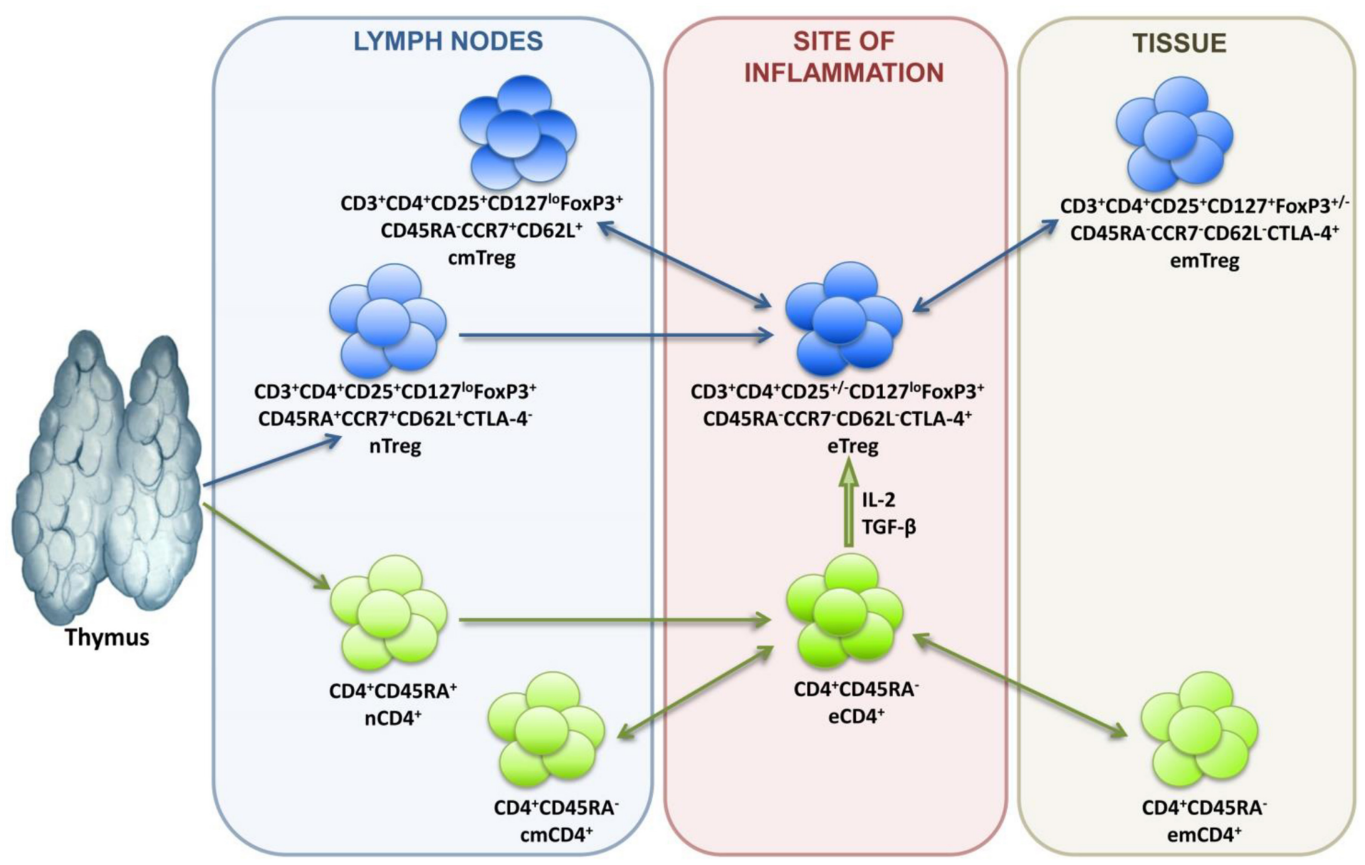
Treg lymphocyte differentiation dynamics.

In terms of differentiation, Tregs are subdivided into naive cells (nTregs), central memory cells (cmTregs), effector memory cells (emTregs), and effector Treg (eTreg) lymphocytes (see Figure 1). CCR7 and CD62L molecules enable Treg homing into the secondary lymphoid organs, while CTLA-4 expression reflects suppressive activity of Tregs. Treg lymphocytes function in different tissues and inflammatory sites, which is why their differentiation is associated with the acquisition of corresponding chemokine receptors and adhesion molecules responsible for directed homing. Thus, CCR4 is for migration to the skin, GPR-15 is for migration to the intestines, and CXCR3, LFA-1, VLA-4, CCR2, CCR5, CCR6, CCR8 are for migration to inflammation zones (47–49).

Transcriptomic analysis of single cells and studying the pathways of Treg differentiation in the transcriptomic space in pseudotime ordering have enabled researchers to study the tissue-specific heterogeneity of the Treg population in detail. They have thus defined the Treg differentiation trajectories in line with their “molecular portraits” and found the percentages of Treg subpopulations in tissues; they have also evaluated the contribution of signaling pathways to maintaining Treg homeostasis (50).

Miragaia et al. assessed the expression of over 30 genes and found that even at their early priming in lymph nodes, Tregs acquire markers of tissue-specific migration. Thus, Tregs that later migrate to the large intestine boost CCR9 and Itga-4 expression (producing integrin-α4), while in mesenteric lymph nodes. Future skin resident Tregs express Cxcr3 and Itgb-1 (producing integrin-β1) genes before migration to the skin, while in shoulder lymph nodes. Thus, the migration properties of T-regulatory cells are already determined in the lymph nodes, although the mechanism for this is unclear (50). This is of particular interest in the context of antigen-specific Treg effects. TCR specificity determines Treg distribution in tissues and lymph nodes; the mechanism behind it is the interaction with dendritic cells in the niche (51–55). Note that after a Treg migrates to a specific tissue, it “matures” or adapts, a process associated with transcriptomic changes (50). At the same time, TCR signal intensity is not related to the Treg activation degree; instead, it determines the phenotype, i.e., Tregs with similar TCR specificity have highly similar transcription profiles (56). In addition, suppressive mechanisms of Tregs have been recently shown to differ if the cells share specificity but differ in TCR affinity. High-affinity receptor cells mostly express TCR-dependent mediators: IL-10, TIGIT, GITR, and CTLA-4; whereas cells having a low-affinity receptor express more Ebi3, which is responsible for IL35-mediated suppressive action. This indicates that affinity determines different functional mechanisms of suppression. In addition to Ebi3, Tregs with low-affinity TCR produce amphiregulin, which is a growth factor that participates in tissue regeneration. Apparently Tregs with low-affinity TCR are more likely to use non-TCR-dependent suppressive mechanisms in the absence of strong TCR signaling in response to humoral inflammatory factors, while high-affinity Tregs preferentially upregulate TCR-dependent regulatory molecules, such as CTLA-4, TIGIT, and IL-10. Nevertheless, both types of cells have suppressive potential and support autotolerance and immune equilibrium (31, 57, 58).

**Figure 2:**
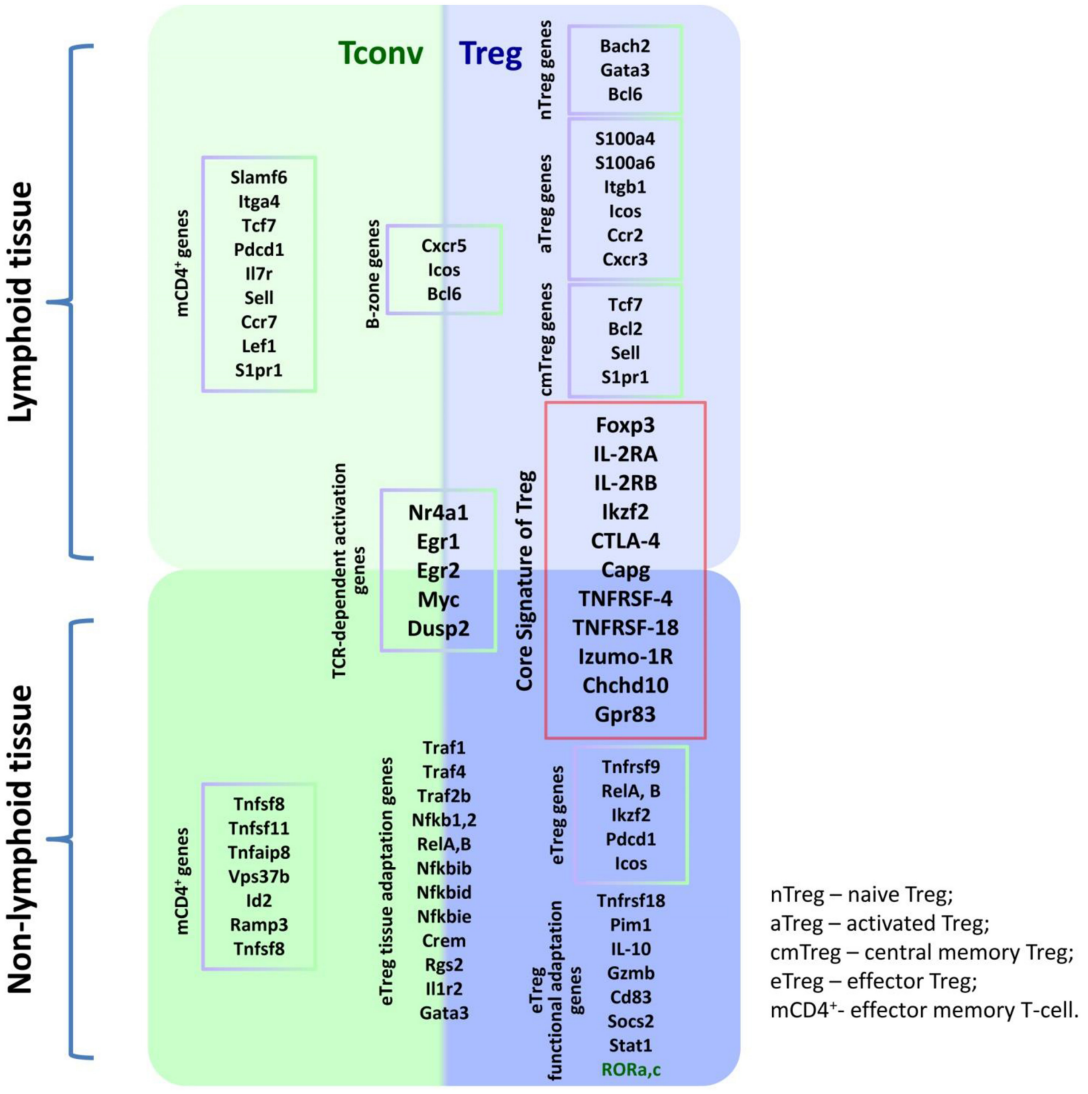
Transcription heterogeneity of the Treg population.

Thus, Treg heterogeneity depends on their origin, differentiation, and migration characteristics, which in turn depend not only on the expression of homing molecules but also on TCR specificity and affinity. Such heterogeneity of the immune response regulating population seems to reflect the diversity of Treg-targeted cells as well as the variety of conditions under which Treg lymphocytes may have to function; in addition, this heterogeneity is associated with the ontogenetic kinship of Tregs and T effectors. Indeed, studies into the Treg/Tconv transcription phenotype identified only a small subset of genes expressed en masse by Tregs that are absent in Tconv. The set is referred to as the Treg signature. Meanwhile, most genes have similar expression profiles in both populations. For instance, the populations overlap in genes whose expression depends on TCR signaling, as well as in genes involved in Treg and Tconv homeostasis in different tissues (50, 56). However, different Treg subpopulations can, in addition to the main signature closely related to the FoxP3 gene, express additional genes responsible for tissue-specific functioning. Research into Treg transcription profiles in a murine spleen identified a common gradient that separates resting and activated Tregs (50), which is largely consistent with the current knowledge of Treg differentiation dynamics (see Figure 1). Unsupervised learning has been employed to identify the clusters of resting nTregs, Tregs in early TCR-dependent activation, and activated Tregs featuring a follicular Treg signature typical of lymph node B zones, various tissues, and sterile inflammation sites (56). Figure 2 presents a simplified diagram of murine Treg transcription heterogeneity in the context of basic signatures and the condition-specific activation of certain genes. It is worth noting that mice and humans have similar Treg transcription profiles (50, 56).

The advancement of single-cell transcriptomics and ML-based multidimensional data clustering opens up great opportunities to study the mechanisms affecting the T cell lifecycle, to analyze the expression of hundreds of genes, and to identify TCR sequences in single cells. This is a great and unprecedented booster for immunology research, one that lays the foundation for potential immunity management.

## Treg Suppressive Activity Mechanisms

In recent years, Tregs have been shown to suppress most immune cell populations, including lymphocytes, various types of macrophages, dendritic cells and B cells (59). Interestingly, the manifestation of Treg suppressive activity against a specific T effector population may be associated with the expression of transcription factors typical of this subpopulation. Thus, the expression of T-bet, a Th-1-associated transcription factor, in Tregs is related to the expression of the inhibitory molecule TIGIT, which binds CD155 to dendritic cells to increase the production of IL-10 and reduce that of IL-12 in the dendritic cell, thus inhibiting the activation of T effectors (60, 61). Tregs with the T-bet^+^TIGIT^+^ phenotype selectively inhibit the Th1- and Th17-mediated proinflammatory immune response (62, 63). Similarly, the Th2-associated transcription factor IRF-4 enables Treg expression of ICOS and CTLA-4; along with JUNB and RBPJ, it is needed to limit the Th2-mediated immune response (64–66). Meanwhile, the expression of the Th17-typical factor STAT3 in Tregs is closely related to the regulation of the Th17-mediated immune response and enables the expression of IL-10, Ebi3, granzyme-B, and perforin-1 genes (67). It is important that the expression of transcription factors typical of this or that subpopulation of effector T lymphocytes may be associated with unstable FoxP3 expression and loss of Treg suppressive functions. This process appears to be greatly correlated with the cytokine background in a Treg microenvironment and manifests itself in pathology (68). Tregs are believed to acquire the expression of transcription factors typical of effector cells owing to their adaptation to the immune response being polarized toward Th1, Th2, or Th17.

The functional heterogeneity of the Treg population reflects the broad spectrum of suppressive mechanisms Tregs use to control various types of immune responses. Those can be conventionally divided into contact and humoral, antigen-specific or non-specific. Many of these mechanisms are versatile and complementary; however, some are specific to certain types of immune responses.

Antigen-specific suppression is mainly caused by the direct Treg–DC (dendritic cell) interaction enabled by the specific recognition of the antigen (Ag) the DC presents as part of MHC-II by means of Treg TCR. Ultimately, such interaction results in inducing an Ag-specific tolerogenic dendritic cell or rendering the DC unable to present a specific antigen. The mechanisms of such suppression are diverse. They include binding the co-stimulation molecules CD80/86 on the dendritic cell by means of CTLA-4 (69); removing Ag-MHC-II from the DC surface by trans-endocytosis, in which case the DC remains capable of presenting other antigens (70–72); and a CTLA-4-mediated increase in IDO expression in the DC, which lowers the concentration of tryptophan necessary for T effectors to proliferate (73). In general, these mechanisms disrupt Ag presentation, cause T effector anergy, or trigger Ag-specific pTreg induction.

Antigen-non-specific mechanisms include the enzymes CD39/CD73 on the Treg surface, which cause ATP to degrade to adenosine. Increased adenosine concentration in the microenvironment inhibits DC presentation of antigens and suppresses the proliferation of activated T effectors (74). Another non-specific suppression factor is the Treg production of cytokines: TGF-β, IL-10, and IL-35. The range of suppressive effects these cytokines have is extremely broad. They can suppress the activation and proliferation of effector T and B lymphocytes; they can also directly induce pTregs and Bregs (75–78). In addition, TGF-β and IL-10 inhibit antigenic presentation to stimulate the generation of tolerogenic dendritic cells, which in their turn enable pTreg induction (79–83). Note that two pTreg populations have been well-described: Th3 and Tr1, featuring high TGF-β, and IL-10 secretion, respectively. The former has been identified by their role in oral tolerance; the latter by their involvement in preventing autoimmune colitis. Both are generated in chronic inflammation sites as well as in transplanted tissue (84).

Tregs have recently been shown to disrupt the Ca2^+^ supply to effector lymphocytes, thus disabling the Ca2^+^-dependent transcription factors NFAT and NF-kB T effectors need in early TCR-dependent activation. This contact suppression mechanism is currently understudied, yet it may play a crucial role in autotolerance (78, 85).

Perforin-granzyme cytolysis is another important contact suppression mechanism characteristic of some subpopulations of activated Tregs. Tregs exhibit perforin-dependent cytotoxicity against a variety of targets, including CD4^+^, and CD8^+^ effector T cells (86).

Tumor necrosis factors also are involved in Treg suppressive functions. As Tregs are activated, they acquire TRAIL (TNF-related apoptosis-inducing ligand) expression, while the CD4^+^ effector cells begin expressing the ligand of this molecule, DR5 (death receptor 5); TRAIL/DR5 interaction induces the apoptosis in effector lymphocytes by activating caspase-8 (87, 88).

Treg lymphocytes simultaneously express the molecule PD-1 and its ligand PD-L1. DC PD-L1 and Treg PD-1 interaction generates a tolerogenic dendritic cell. Treg PD-L1 interacts with PD-1 on the activated effector cells and causes its anergy or even induces the pTreg. In such interaction, the signal is transmitted to the same Treg. The PD-1 transmitted signal is crucial to FoxP3 expression and for maintaining Treg homeostasis. The mechanism is not Treg-specific; it is also important for carcinogenesis and tumor evasion of CD8^+^ and NK lymphocytes (89).

Because of high IL-2R (CD25) expression, Tregs can reduce IL-2 concentrations in the microenvironment, which will negatively affect the proliferative response of CD8^+^ cells. The mechanism seems less significant for suppressing the proliferation of CD4^+^ lymphocytes, which is due to CD4^+^ and CD8^+^ differing in their sensitivity to IL-2 (90).

Treg suppressive mechanisms are currently understudied. For example, Tregs expressing HLA-DR manifest early contact suppressive activity associated with high FoxP3 expression. Such HLA-DR^+^Treg lymphocytes are mature Treg effector cells. At the same time, the antibody blockade of the molecule HLA-DR causes Tregs to lose their suppressive activity *in vitro* (91). However, the antigen specificity of such suppression remains an unanswered question. Which role MHC-II has to play in this process and how the expression of these molecules emerges on the Treg surface remains to be seen. Some suggest this occurs as part of transendocytosis in Treg-DC interaction, meaning that a Treg is capable of direct antigen-specific contact suppression. However, this conclusion may be premature because this question requires further research (92).

**Figure 3:**
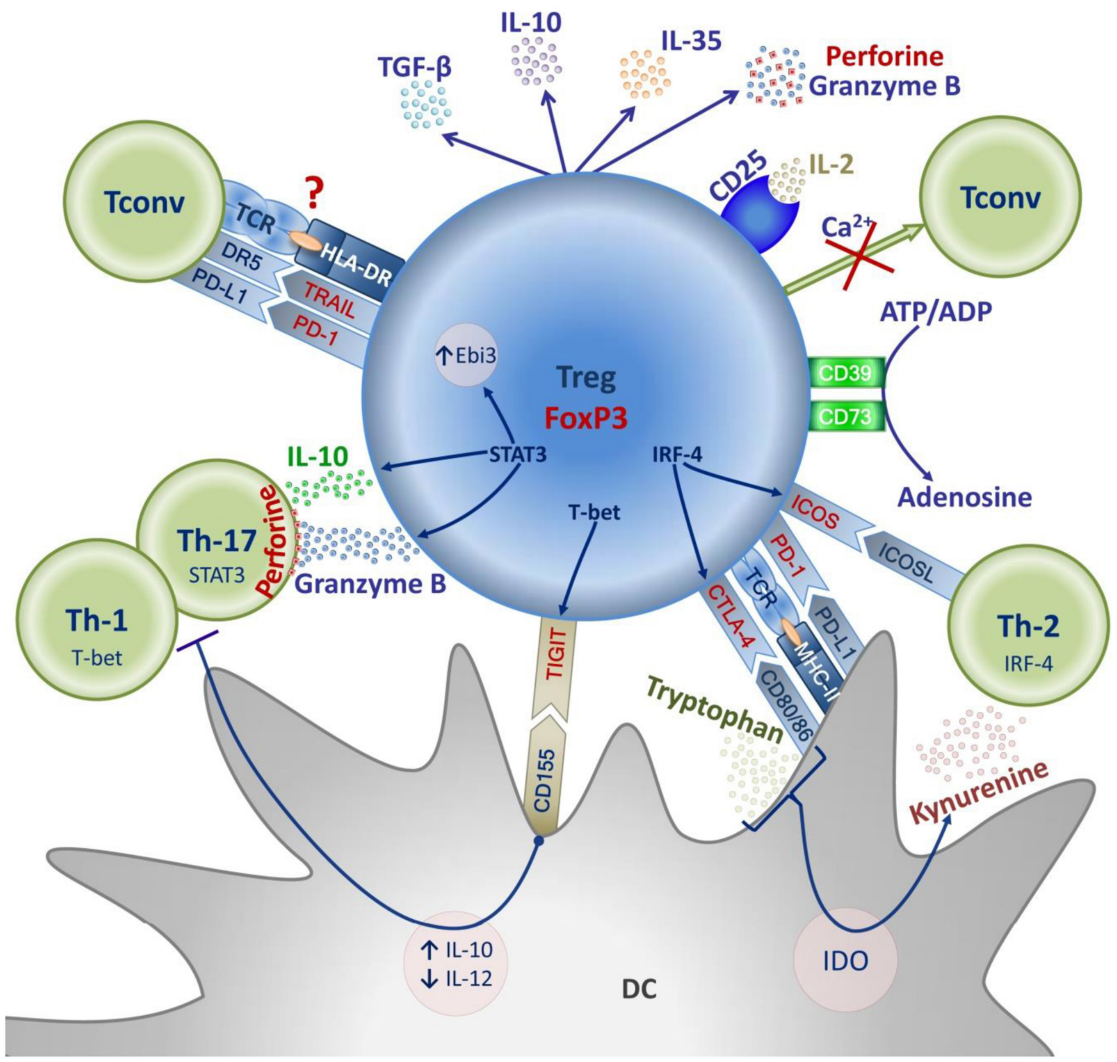
Mechanisms of Treg suppression.

The described mechanisms of Treg-mediated suppression are summarized in Figure 3.

It is worth noting that, in addition to suppressive functions, Tregs regulate tissue repair and regeneration. Tregs interact with innate and adaptive immune cells and regulate their activities after tissue injury. Tregs are involved in tissue-specific repair of the muscle, bone, lung, skin and central nervous system (93). The mechanisms of action may vary from tissue to tissue and include: amphiregulin (a growth factor) production, promotion of proliferation and differentiation of stem cells of different tissues, inhibition of neutrophil extravasation and monocyte activity and also the limitation of osteoclastogenesis (93, 94). In addition, the finding of Rieckmann et al. seems to be an important discovery in the context of TCR Treg specificity. They recently demonstrated that the epitope of myosin heavy chain α is a dominant cardiac antigen triggering CD4^+^ T cell activation after myocardial infarction in mice. Such Ag-specific T cells selectively accumulated in the myocardium and mediastinal lymph nodes of infarcted mice, acquired a Treg phenotype with a distinct prohealing gene expression profile, and mediated cardioprotection (95). Thus, it seems to be important to investigate new strategies in Treg-mediated tissue regeneration while considering TCR specificity for potential clinical use.

## Treg Homeostasis

**Figure 4:**
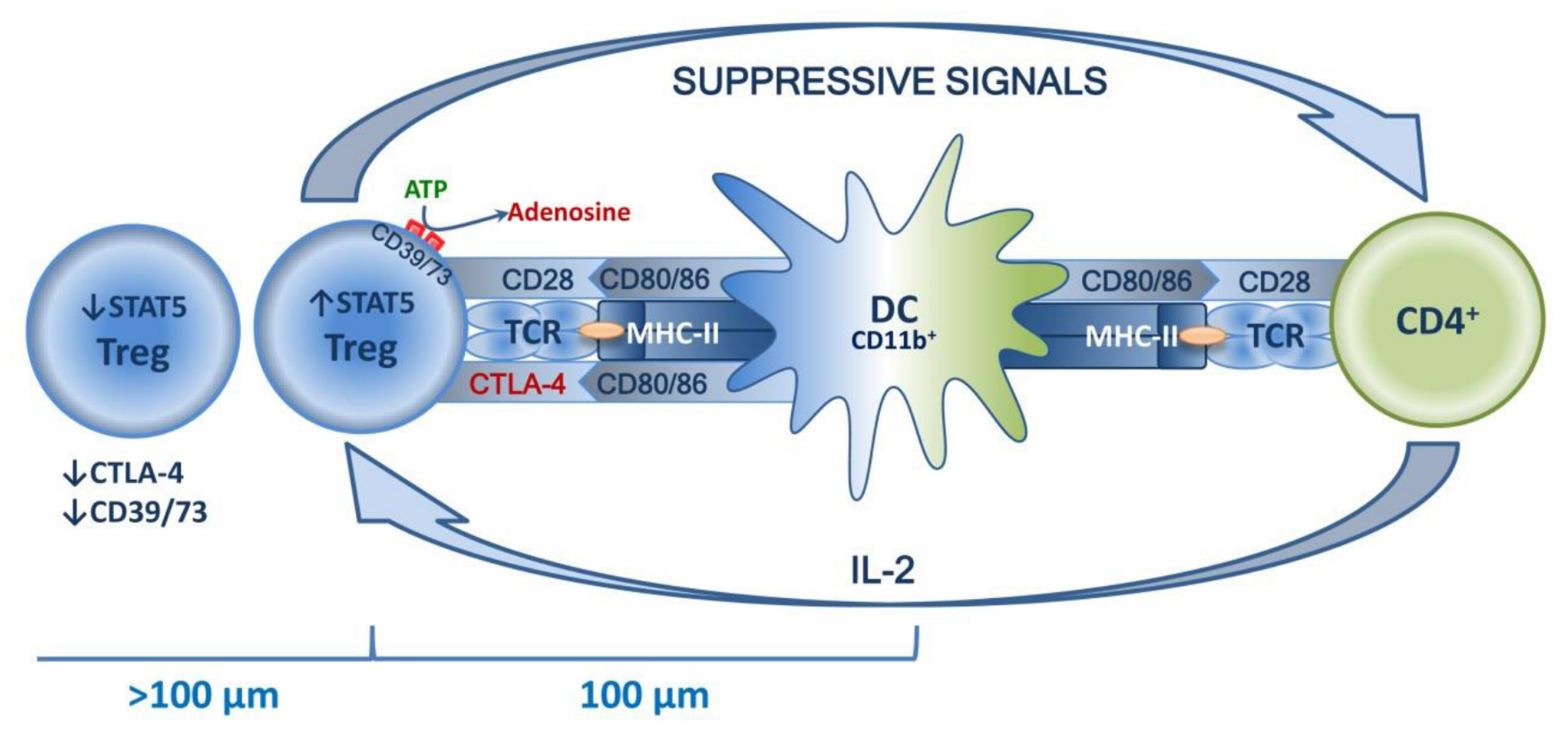
Schematic presentation of a Treg niche.

The common mechanisms of Treg homeostasis are well-known. The main mechanisms are a continuous subliminal signal from TCR, which recognizes the autopeptide in MHC-II (96, 97); co-stimulation signals, in particular those mediated by CD28 in contact with CD80/86 (98, 99); and the effects of humoral factors, primarily IL-2 (100, 101). The combined effects of these factors are observed in the special niches located in the T zones of secondary lymphoid organs, and the effects are DC-mediated. Distribution in niches is tissue-specific and is enabled by TCR affinity to corresponding peptides, as well as by Tregs acquiring characteristic homing molecules as they mature (52–54). Typically, draining lymph nodes function as secondary lymphoid organs. These are key sites for priming the autoreactive T cells, and it seems that they are involved in both inducing and suppressing the tissue-specific immune response (51). This indicates that dLN are the key “arena” of various homeostatic forces, the result of which determines the balance of tolerance and the immune/autoimmune response. This niche theory seemed to be proven by the recent research of Liu et al., who have shown that in secondary lymphoid organs, highly suppressive Tregs are localized in separate clusters and surround autoreactive lymphocytes. These lymphocytes are dominated by activated CD4^+^ cells that feature a high production of IL-2, which is necessary for Tregs to function. The central place in such clusters is taken by mature DCs that feature high expression of MHC-II and co-stimulatory molecules, mostly of the CD11b^+^ phenotype. At the same time, Tregs closer to the center express significantly more STAT5 in addition to CTLA-4 and CD73. Further from the center (>100 μm), Tregs have their STAT5, CTLA-4, and CD73 expression drastically reduced, which is due to a lower IL-2 concentration further away from the cluster center. T effectors and their associated increase in IL-2 production trigger compensatory Treg activation, increasing their suppressive activity. In turn, loss of TCR signal or antibody blockade of IL-2 deforms the cluster, which has negative implications for Treg functioning and is accompanied by excessive activation of effector T lymphocytes (102). Thus, autotolerance maintenance is an active process based on subtle feedback regulatory mechanisms implemented in peripheral lymphoid organs at a cluster level involving DCs, Tregs, and effector T lymphocytes (see Figure 4).

A number of studies prove the concept presented above. Animal experiments have shown the direct correlation of the peripheral Treg pool with the homeostatic mechanisms above. In transgenic mice, depleting the pool of DCs presenting a specific antigen causes a terminal reduction in the population of Treg clones of specific TCR specificity; increasing this DC pool will dramatically raise the Treg clone population (103, 104). *In vivo* and *in vitro* experiments show that disrupting the CD28 and CD80/86 interaction cuts the Treg population and reduces CD25 expression (105–107). In addition, experiments with transgenic mice have revealed a very important pattern: the peripheral Treg pool is a function of the number of effectors capable of IL-2 production (108). Recent research shows that DCs regulate the population and suppressive activity of Tregs by means of the Lkb1-kinase. Knockout of the gene Lkb1 in DCs causes excessive Treg expansion in various organs, deteriorating the antigen-specific immune response. The mechanism behind this phenomenon is associated with increased OX40L expression on DCs, which in turn is caused by activating the nuclear factor NF-kB as Lkb1 activity lowers. The interaction of Treg OX40 and DC OX40L boosts Treg proliferation and suppressive activity. In doing so, researchers discovered that administering LPS to wild mice selectively inhibited Lkb1 expression in DCs and expanded the Tregs while increasing the expression of proinflammatory genes in DCs, which was further confirmed by testing the transcription profile of such DCs. The DC thus remains capable of antigen presentation and priming T effectors. Therefore, Lkb1 may constitute an important factor that DCs use to regulate the immune response. Chen et al. note that the above study calls into question the concept of regulatory DCs; it proves the co-execution of regulatory and inflammatory programs controlled by various signals in the same DC as it is activated and matures (109). More research is necessary to study the mechanisms of controlling Lkb1 expression in DCs, as well as the possible association of changes in the activity of this kinase in various DC types when exposed to different autoimmune diseases or tumors.

Depending on the maturity and tissue distribution, Treg homeostasis may be based on different mechanisms. Just like IL-2, IL-7 is crucial for nTregs circulating between secondary lymphoid organs. This cytokine is produced in lymph nodes by reticuloendothelial cells and enables nTregs to survive and proliferate by increasing the expression of Bcl-2 and Ki-67. As with IL-2, the IL-7 effects are mediated through JAK3/STAT5 and PI3K/Akt signaling pathways (110). It is worth noting that thymopoiesis has quite a low impact on maintaining the nTreg pool in humans, which is confirmed by the minimal effects of age involution and early thymectomy on the nTreg population numbers in adults despite the loss of thymic output and the decrease in the nTreg number at the early stage after thymectomy (110, 111). This is caused by the compensatory peripheral proliferation of naive Treg cells, resulting in the maintenance of the nTreg population. Similar homeostatic maintenance mechanisms are known for cmTreg lymphocytes, as well. Like nTregs, the cells of this weakly proliferating population circulate between secondary lymphoid organs and express anti-apoptotic factors Bcl-2 and Mcl-1 at a high level. IL-2, constitutively produced by Tconv in the lymph node niche, is an important homeostatic factor for these cells (112, 113). Conditions required to maintain Treg memory cell homeostasis have not been studied in such detail because they are tissue-specific, while the emergence of each population invokes multiple mechanisms that vary from location to location. These are long-lived cells emerging from effector Treg lymphocytes after preventing or resolving a primary inflammation; they have a stronger suppressive effect on an immune response caused by secondary contact with a corresponding antigen (114). Experimenting on transgenic mice has shown that differentiation of regulatory memory cells required IL-2 while maintaining a population of these cells required IL-7 but not IL-2. This is reflected by the phenotype of this Treg population because they feature a high expression of IL-2 and IL-7 receptors (CD25^+^CD127^+^) (47). Effector Treg lymphocytes are more complicated because they function in different organs and tissues, different inflammation sites, and in the corresponding draining lymph nodes. These cells proliferate actively and are prone to apoptosis because of low Bcl-2 and Mcl-1 expression (112). Their homeostasis is not as dependent on IL-2 and IL-7; rather, it relies on a strong TCR signal that enables the expression of eTreg-specific genes (115). Given that selective Treg effects on different immune response types require different stimuli and are associated with specific transcription changes, drawing a clear border between homeostatic signals and differentiation signals for these cells is difficult.

IL-12 and IFNγ are the main humoral factors typical of the Th1-mediated immune response. However, only the latter is involved in the maturation of Th1-specific Tregs. Binding to IFNγR receptor on Tregs, IFNγ activates STAT1 and triggers T-bet expression, which causes CXCR3 to be expressed and Tregs to migrate to the sites of Th1-mediated inflammation (116). Fluorescent visualization has shown that CXCR3^+^(T-bet)^+^Tregs are far closer to T-bet^+^Th1 and CD8^+^ lymphocytes than CXCR3^−^(T-bet)^−^Tregs, which indicates the lower suppressive activity of CXCR3^−^(T-bet)^−^Tregs with respect to the Th1-mediated immune response. Furthermore, analysis into the TCR repertoire has revealed that CXCR3^+^(T-bet)^+^Tregs and CXCR3^−^(T-bet)^−^Tregs differ in antigenic specificity, which also reflects the functional difference between these subpopulations (62). It is worth noting that T-bet expression is several times lower in Tregs than in Th1 lymphocytes, while the high TCR-dependent expression of FoxP3 suppresses the activation of T-bet-dependent proinflammatory genes in Tregs, thus preventing Tregs from transforming into Th1. Therefore, TCR and IFNγ signals determine the functional maturation and homeostasis of effector Tregs in the context of the Th1-mediated immune response.

eTreg effects on other types of immune responses are less specific and are associated with the TCR-dependent activation of the transcription factor IRF4, which is required for Tregs to function. At the same time, disabling the gene IRF4 in murine Tregs results in an autoimmune syndrome mainly mediated by activating the Th2 type of immune response (64). The expression of this factor is also observed in various populations of effector T cells: Th2, Th9, Th17, Tfh, to which the effector IRF4^+^Treg can be suppressive (117). IRF4 effects in Tregs are mediated by the transcription factor JunB, which binds IRF4 to target genes in the DNA, thus contributing to the expression of effector Treg molecules such as ICOS and CTLA-4 (66). The expression of ICOS on eTregs is of extreme importance because when interacting with its ligand ICOS-L, this molecule enables Tregs to survive and suppress more efficiently. The mechanism behind ICOS is associated with NFAT activation, which boosts the transcription activity of FoxP3 that, in its turn, enables the expression of the genes IL-4, IL-10, and TGF-β. In addition, the ICOS signal further activates protein kinase B (Akt), which is crucial to Treg survival because it inhibits Treg apoptosis (118). The significance of ICOS in maintaining the homeostasis and functional activity of Tregs has been confirmed by multiple studies. Thus, the lack of expression of this molecule in mice is associated with CNS2 hypermethylation and loss of FoxP3 expression, as well as with a 30% reduction in the Treg pool (119); the antibody blockade of this molecule disrupts Treg functioning (120, 121). Note that the ligand ICOS-L is expressed on the DC; a lack of it may also result in a loss of Treg functionality. Thus, when macroautophagy processes in the DC are disrupted, there accumulates metalloproteinase, which is involved in ICOS-L breakdown. Disrupting the expression of this molecule on DCs negatively affects the suppressive function and stability of Tregs (122). Thus, a strong TCR signal and co-stimulator signals from DCs transmitted from ICOS/ICOS-L are key to the functional activity and homeostasis of the effector eTreg lymphocytes mainly involved in regulating the Th2-mediated immune response.

To act on Th17, eTregs require activating the factor STAT3, which enables selective suppression of the Th17-mediated immune response (see above) (67). STAT3 is activated by various cytokines, including IL-6, and IL-23, which are typical humoral factors of the Th17 immune response. However, STAT3 is only activated in Tregs by means of IL-10, whereas a disrupted expression of IL-10R, as well as disabling the expression of STAT3 in Tregs, accelerates the Th17 response and triggers severe intestinal inflammation (67, 123). IL-10 effects seem to be the most important homeostatic factor that keeps Tregs functional with respect to the Th17-mediated immune response. The effects of this factor are apparently paracrine. It appears that in the case of the Th17-mediated immune response associated with high concentrations of proinflammatory cytokines that negatively affect the stability of FoxP3 expression, the paracrine effects of IL-10 may not suffice to stabilize FoxP3 expression. In that case, Tregs transform into exFoxP3Th17 lymphocytes. This is what frequently accompanies autoimmune diseases such as rheumatoid arthritis.

All of the abovementioned studies prove the paramount role of dendritic cells, co-stimulatory and humoral factors in Treg homeostasis. The relative contribution of these mechanisms to maintaining the homeostasis of different Treg populations may vary from setting to setting. For instance, an immune response, lymphopenia, or inflammation, as well as the emergence of a tumor, may trigger additional contextual mechanisms. Still, they are generally all aimed at enabling the expression of genes of the main Treg signature, as well as that of genes associated with functional adaptation to such conditions; this is indicated by the transcription and functional heterogeneity of Treg populations (50, 56) (Figure 2).

## Conclusions

Intensive research in recent decades has described the population of Treg lymphocytes as a separate T cell subpopulation mainly designed to selectively regulate immune response while maintaining autotolerance. Considering selectivity, a basic property of Tregs helps address a contradiction that exists in one of the general autoimmune disease and tumor pathogenesis theories. According to this theory, cancers are triggered by excessive Treg activity while autoimmune processes are due to too low activity. What makes it contradictory is that cancers and autoimmune pathologies may well be concurrent. Given that Tregs are selective to various types of immune responses, and such selectivity depends on antigenic stimuli and the spatial localization of the process, it may be assumed that pathology is caused by the failure of a specific Treg cluster that features a common TCR repertoire of similar antigenic specificity. Ever more data are collected that prove the role TCR plays not only in maintaining the homeostasis, or activation, of Tregs but also in the phenotypic selection because the antigenic specificity of TCR determines the type of immune response Tregs regulate. This is confirmed by the difference in location and transcription profiles of Tregs that differ in TCR specificity (56, 62). This all indicates a clonal organization of the general Treg pool, an organization based on the close antigenic specificity of TCR in the same clone. Indirect evidence of this concept are the positive results obtained *in vivo* using Tregs with a chimeric antigen receptor (CAR) to treat autoimmune diseases as well as in transplantation. Such CAR-Tregs specifically migrate to target sites and exhibit more pronounced antigen-specific suppressive activity (124), thus filling a gap in a concrete set of Treg clones specific to antigenic determinants with respect to which tolerance is impaired.

A rising Treg population in a tumor positively correlates with disease progression and low survival rates in cancer patients. Research has shown that the effects of Tregs in different tumors are also antigen-specific, which determines the activation and expansion of certain Treg clones in the tumor microenvironment (125). Perhaps novel approaches based on selective suppression of antigen-specific tumor Treg clones will yield better results than using monoclonal antibodies as functional markers characteristic of the general pool of effector Tregs such as PD-1/PD-L1 or CTLA-4.

One additional important piece of evidence of the role of the Ag-specific clonal organization of Treg populations in their functioning was obtained by Bacher's team in 2016. They showed that aeroallergen allergy development linked with imbalance between Ag-specific Treg and Th-2 lymphocytes, which have a specificity of TCR to a narrow set of epitopes. Herewith, such an imbalance was revealed to be associated with the physical entity of these epitopes. These epitopes, which quickly pass into soluble form, predominantly activate Th-2 cells, while particle proteins activate and apparently stimulate proliferation predominantly of Treg cells. This leads to activation of Treg and Th-2 clones with a non-overlapping repertoire of TCR. Thus, a common pool of Treg cells and other Ag-specific Treg clones retain functionality and numbers as in healthy individuals, or have increased functional activity. That demonstrates qualitative and quantitative preservation of a common Treg pool in allergic individuals. This finding highlights necessity to induce Ag-specific Treg responses rather than rely on strategies aimed at activating the existing Treg pool (126).

The negative impact of homeostatic proliferation on the Tconv population leads to changes in the TCR landscape, such as a decrease in the TCR repertoire diversity and oligoclonal expansion (127, 128). Therefore, it seems to be important to investigate the influence of homeostatic proliferation on the Treg pool in the context of clonal organization according to TCR specificity. Because, aside from quantitative maintenance of the nTreg pool, it is important to consider qualitative changes in TCR Treg diversity, which may occur during the homeostatic proliferation of Treg cells with age, and may affect the immune equilibrium in older adults by forming some gaps in the landscape of the naïve Treg TCR repertoire.

Summarizing the data from this review, we conclude that TCR specificity and affinity not only play a key role in thymic selection and maturation of Treg cells but also in determining the further fate of these cells, governing tissue-specific distribution, transcriptomic profile and ultimately determining participation in a particular type of immune response.

In recent years, immunology has seen great advances in its methodologies. New techniques have appeared that enable genomic and transcriptomic analysis, allowing researchers to evaluate the expression of dozens of different proteins in single cells; for example, machine learning-based data mining has become a reality. All of this opens up ample opportunities to research cellular interactions and lays foundations for fundamentally new approaches to the treatment of various diseases by using targeted antigen-specific effects on various components of the immune system to restore the once disturbed equilibrium of the immunity.

## Author Contributions

DS contributed to the conception, drafting of the manuscript and design. VT contributed to the conception, revision, and final approval of the manuscript.

### Conflict of Interest

The authors declare that the research was conducted in the absence of any commercial or financial relationships that could be construed as a potential conflict of interest.
